# LcMPK3 and LcMPK6 positively regulate fruitlet abscission in litchi

**DOI:** 10.1186/s43897-024-00109-z

**Published:** 2024-08-06

**Authors:** Fei Wang, Zhijian Liang, Xingshuai Ma, Zidi He, Jianguo Li, Minglei Zhao

**Affiliations:** 1 State Key Laboratory for Conservation and Utilization of Subtropical Agro-Bioresources, South China Agricultural University, Guangzhou, 510642 China; 2 Key Laboratory of Biology and Genetic Improvement of Horticultural Crops (South China), Ministry of Agriculture and Rural Affairs, South China Agricultural University, Guangzhou, 510642 China; 3 Guangdong Litchi Engineering Research Center, College of Horticulture, South China Agricultural University, Guangzhou, 510642 China

**Keywords:** Litchi, Fruit abscission, MPK3/6

## Abstract

Mitogen-activated protein kinase (MAPK) cascades have been discovered to play a fundamental role in regulating organ abscission. However, the identity of protein substrates targeted by MAPK cascades, as well as whether the role of MAPK protein cascades in the abscission process is conserved across different plant species, remain unknown. Here, the role of homologs of MPK3 and MPK6 in regulating fruit abscission were characterized in litchi. Ectopic expression of *LcMPK3* or *LcMPK6* in *Arabidopsis mpk3 mpk6* mutant rescued the deficiency in floral organ abscission, while silencing of *LcMPK3* or *LcMPK6* in litchi significantly decreased fruitlet abscission. Importantly, a total of 49 proteins interacting with LcMPK3 were identified through yeast two-hybrid screening, including two components of the MAPK signaling cascade, five transcription factors, and two aquaporins. Furthermore, the interaction between LcMPK3/6 with LcBZR1/2, core components in brassinosteroids signaling that suppress litchi fruitlet abscission, was confirmed using in vitro and in vivo assays. Moreover, phos-tag assays demonstrated that LcMPK3/6 could phosphorylate LcBZR1/2, with several phosphorylation residues identified. Together, our findings suggest that LcMPK3 and LcMPK6 play a positive regulatory role in fruitlet abscission in litchi, and offer crucial information for the investigation of mechanisms underlying MPK3/6-mediated organ abscission in plants.

**Supplementary Information:**

The online version contains supplementary material available at 10.1186/s43897-024-00109-z.

## Core

Silencing of LcMPK3 or LcMPK6 in litchi decreased fruitlet abscission. 49 proteins interacting with LcMPK3 were identified. The interaction between LcMPK3/6 and LcBZR1/2, core components in brassinosteroids signaling that suppress fruitlet abscission, was confirmed. Phos-tag assays demonstrated that LcMPK3/6 could phosphorylate LcBZR1/2.

## Gene and accession numbers

Sequence data from this article can be found in the litchi genome database (http://www.sapindaceae.com/GeneSearch/GeneSearch.html) under the accession numbers: LcMPK3: LITCHI024767, LcMPK6: LITCHI014949, LcBZR1: LITCHI006688, LcBZR2: LITCHI010361, LcPIP2;5: LITCHI027285, LcZFP1;1: LITCHI018676.

## Introduction

Abscission in plants involves cell separation that enables the drop of no longer needed organs such as infected or non-functional leaves, flowers, and fruits. Abscission in plants takes place in a specialized area known as the abscission zone (AZ), and it is a complex and intricately orchestrated physiological event (Tranbarger and Tadeo 2020). Typically, abscission encompasses four distinct phases: (a) the formation of the AZ at the location where the organ will eventually detach; (b) the development of sensitivity in AZ cells to signals that initiate abscission; (c) the initiation of the detachment process within the AZ, leading to organ separation; and (d) the formation of a protective barrier on the plant’s side of the severed surface (Estornell et al. 2013). Abscission serves as a crucial regulatory process for plants to cope with environmental conditions and ensure the survival of their progeny. However, untimely abscission can lead to substantial economic losses, such as the premature shedding of reproductive structures, which diminishes fruit yield and agricultural output. Conversely, excessive crop load, constrained by resource availability, can degrade the quality of the produce. Consequently, gaining insight into the mechanisms of abscission is vital for enhancing crop breeding and farming techniques, aiming to achieve an ideal balance in crop density.

Significant advancements have been made in elucidating the molecular components that govern the abscission process. Notably, a specific signaling pathway has been identified as pivotal in controlling the onset of floral organ abscission in *Arabidopsis*. Two decades ago, Butenko et al. (2003) made a groundbreaking discovery, identifying IDA (INFLORESCENCE DEFICIENT IN ABSCISSION) as a central regulator in this process. IDA, which encodes a small secreted protein acting as a ligand, is crucial for floral organ abscission, with mutations leading to a deficiency in this process. Further research uncovered the role of HAESA (HAE) and HAESA-LIKE2 (HSL2), a pair of closely related leucine-rich repeat receptor-like kinases, which redundantly regulate the abscission of floral organs (Cho et al. 2008; Jinn et al. 2000; Stenvik et al. 2008). Intriguingly, IDA serves as the ligand for HAE/HSL2, initiating a signaling cascade that includes the MAPK pathway involving MKK4/MKK5 and MPK3/MPK6, as well as the transcription factors BREVIPEDICELLUS (BP) and AGAMOUS-like 15 (AGL15) in *Arabidopsis* (Cho et al. 2008; Shi et al. 2011; Patharkar and Walker 2015). In recent years, orthologs of IDA and HAE/HSL2 have been identified across a range of crop species, such as tomato (Tucker and Yang 2012; Li et al., 2021; Lu et al. 2023), soybean (Liu et al. 2018), yellow lupine (Wilmowicz et al. 2018, 2021), rose (Singh et al. 2023), and woody fruit crops like oil palm (Sto et al. 2015), citrus (Estornell et al. 2015), and litchi (Ying et al. 2016; Wang et al. 2019). However, the conservation of the IDA-HAE/HSL2 signaling pathway across different plant species remains an open question that warrants further investigation.

MAPK cascades play a crucial role in eukaryotic organisms, acting as signaling modules that respond to environmental stimuli and regulate growth, development, and adaptation (Zhang and Zhang 2022). These pathways typically consist of a series of three kinases: a MAPK (MPK), a MAPK kinase (MAPKK, also known as MKK or MEK), and a MAPKK kinase (MAPKKK, also known as MKKK or MEKK). Often, there are multiple kinases at each level, which may have overlapping or complementary roles, enhancing the complexity of the signaling network. Upon stimulation, the activation of the initial MAPKKK initiates a phosphorylation cascade, activating the subsequent MAPKKs and ultimately the MAPKs. The activated MAPKs then phosphorylate various targets to trigger cellular responses (Suarez-Rodriguez et al. 2007; Colcombet and Hirt 2008; Wang et al. 2020; Zhang and Zhang 2022). It has established that the MKK4/MKK5-MPK3/MPK6 module plays a critical role in activating the floral organ abscission in *Arabidopsis* (Cho et al. 2008). The double RNAi suppression of *MKK4* and *MKK5*, along with the overexpression of dominant-negative *MPK6* variants in an *mpk3* mutant background, results in abscission phenotypes similar to those of *hae hsl2* and *ida* mutants (Cho et al. 2008). However, to our knowledge, apart from the reported upregulation of *LiMPK6* during the flower shedding process in lotus flowers (Wilmowicz et al. 2021), there have been no reports of homologous genes of MAPK being involved in plant organ abscission.

The phosphorylation of transcription factors is an important mechanism of action for MAPK signaling. In *Arabidopsis*, the MKK9-MPK3/6 cascade has been shown to phosphorylate threonine 173 of EIN3, enhancing the stability of the EIN3 protein and thereby playing a significant role in the regulation of ethylene signaling (Yoo et al. 2008). Recent studies have highlighted the involvement of MAPK-transcription factor (MAPK-TF) modules in the ripening of fruit. In apple, the MdMPK4-14G positively regulates fruit skin chlorosis by phosphorylating the MdERF17 at threonine 67 (Wang et al. 2022). Additionally, ethylene facilitates the phosphorylation of the transcription factor MdNAC72 by MdMAPK3, which promotes fruit softening (Wei et al. 2023). In strawberry, the NAC transcription factor RIF, which is involved in ripening induction, interacts with and is phosphorylated by FvMAPK6 at threonine 310. This phosphorylation event modulates the transcriptional activation function of FvRIF, thereby controlling strawberry fruit ripening (Li et al. 2023). In citrus, CsMPK6 phosphorylates the transcription factor CsMYC2, reducing its promoter-binding activity and stability. This leads to a decrease in jasmonate-induced carotenoid accumulation and a reduction in fruit coloration (Yue et al. 2023). Regarding the abscission process in *Arabidopsis*, although AGL15 has been observed to be differentially phosphorylated in floral receptacles in a MKK4/5-dependent manner, the direct phosphorylation of AGL15 by MKK4/5 has not been confirmed (Patharkar and Walker 2015). In other words, the precise proteins phosphorylated by MAPKs during abscission and the universality of the role of MAPK signaling cascades in abscission across various plant species remain to be elucidated.

*Litchi chinensis* Sonn., a highly popular tropical and subtropical fruit in the global market, originated from Yunnan, China, and is now grown in over 20 countries due to its unique flavor and attractive fruit color (Hu et al. 2022). However, litchi is prone to fruit drop during its development, with 3–5 waves of fruit drop occurring depending on the cultivar, making it an excellent candidate for investigating the underlying molecular processes (Zhao and Li 2020). Our previous studies have identified crucial genes within the IDA-HAE/HSL2 signaling pathway that play a role in litchi fruitlet abscission (Ying et al. 2016; Wang et al. 2019). Importantly, we have revealed that the initiation of litchi fruitlet abscission is tightly regulated by the interplay between ethylene and auxin signaling in the abscission zone (Ma et al. 2020, 2023; Zhao et al. 2020; Ma et al. 2021a, b). Most recently, it has been discovered that the transcriptional control of the LcIDL1–LcHSL2 complex by the auxin response factor LcARF5 integrates auxin and ethylene signaling for litchi fruitlet abscission (Ma et al. 2024). In this research, we discovered that the MPK3/6 homologs in litchi, LcMPK3/6, exert a positive influence on the abscission of fruitlet. Notably, our investigation identified proteins interacting with LcMPK3 and LcMPK6 via yeast two-hybrid screening, including potential candidates like LcBZR1/2, essential core transcription factors in brassinosteroid signaling to inhibit fruitlet abscission in litchi. These findings offer valuable insights for delving into the mechanisms through which LcMPK3/6 govern fruit abscission processes in litchi.

## Results

### Identification of LcMPK3 and LcMPK6 in *litchi*

**Fig. 1:**
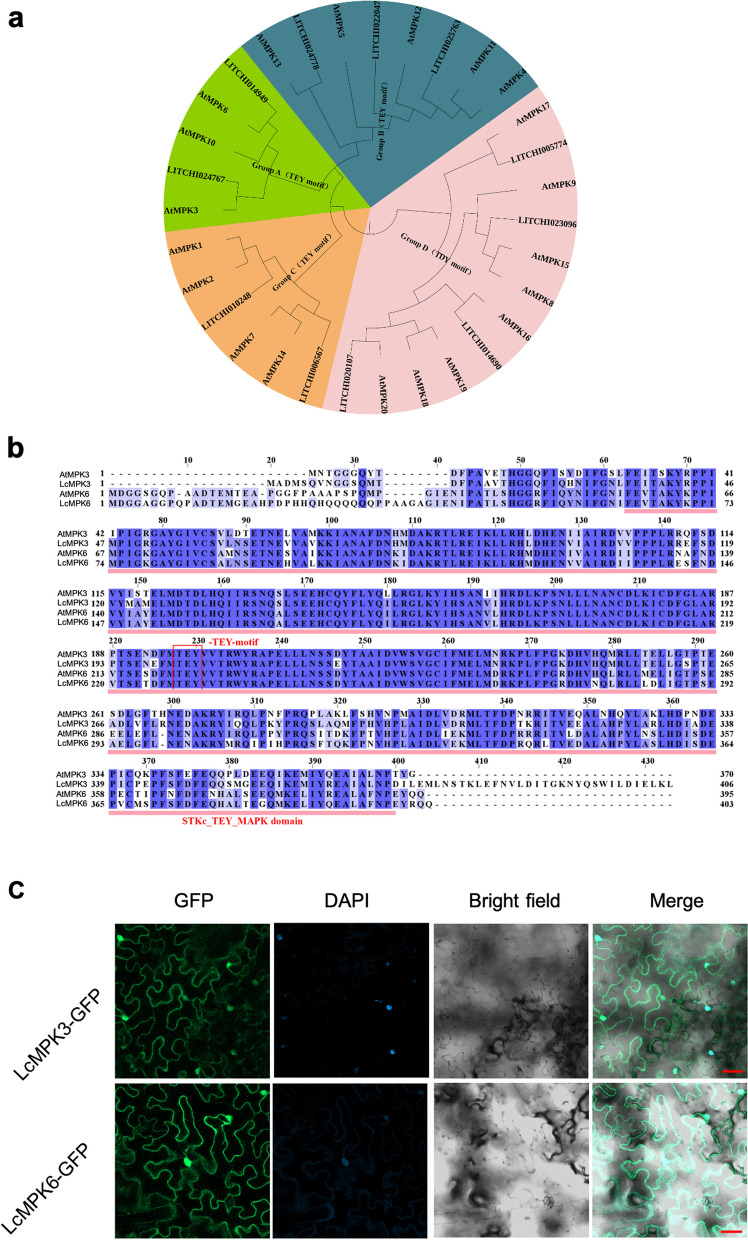
Sequence analysis and subcellular localization of LcMPK3/6. **a** The phylogenetic analysis of LcMPKs in relation to *Arabidopsis* MPKs. **b** The multiple sequence alignment of LcMPKs with *Arabidopsis* MPKs is presented, with the TEY motif and STKc_TEY_MAPK domain, which are hallmarks of MPK proteins, highlighted by a red box and red underline, respectively. **c** The subcellular localization of LcMPK3/6 in *Nicotiana benthamiana* leaves is shown. After a 48-h incubation period, the GFP and nuclear marker DAPI signals were visualized using confocal laser scanning microscopy (CLSM), with scale bars representing 50 μm

Recognizing the significance of MPK3 and MPK6 in *Arabidopsis* for controlling organ shedding, we utilized the amino acid sequences of these *Arabidopsis* kinases to conduct a TBLASTN search within the litchi genome. This approach led to the identification of 11 LcMPKs, which are distributed across eight chromosomes (Figure S1). Within the *Arabidopsis* genome, a total of 20 MAPKs have been identified. These MAPKs are readily distinguishable due to their sequence homology and the distinctive TX (E or D) Y activation motif. The activation of MAPKs occurs upon dual phosphorylation of the Thr and Tyr residues within this motif by upstream MAPKKs (Zhang and Zhang 2022). A phylogenetic analysis revealed that MAPKs can be classified into four groups (A, B, C, and D). Two litchi MAPKs proteins, which were divided into grouped A, were closely related to *Arabidopsis* MPK3 and MPK6, and were consequently named LcMPK3 (LITCHI024767) and LcMPK6 (LITCHI014949) (Fig. 1a). Sequence alignment of these proteins indicated the presence of a conserved TEY motif and a conserved STKc_TEY_MAPK domain (Fig. 1b).

**Fig. 2:**
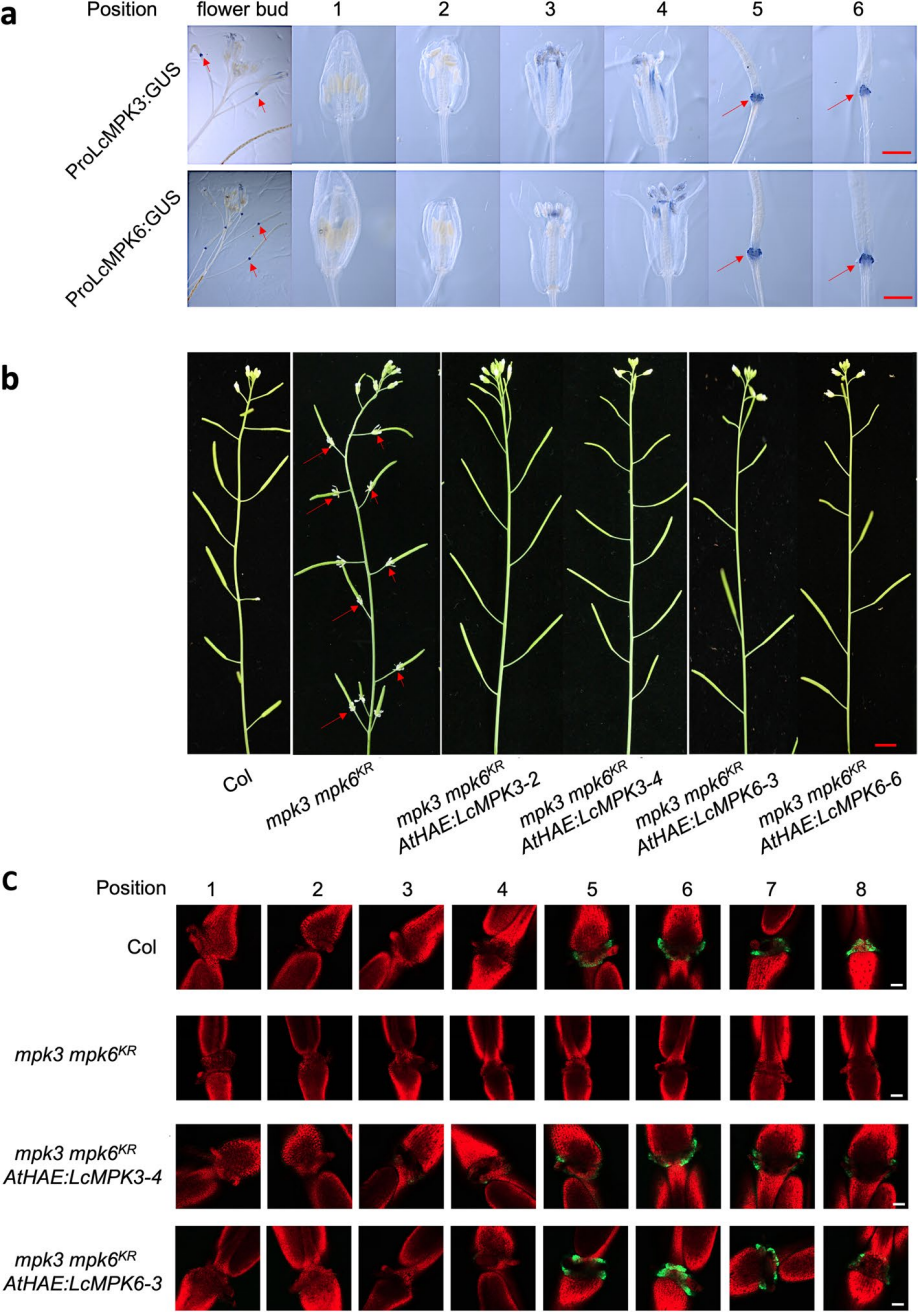
LcMPK3/6 exhibit conserved roles in activating floral organ abscission in *Arabidopsis*. **a** GUS activity, driven by the promoters of *LcMPK3* or *LcMPK6*, is observed at the floral organ abscission zone (AZ) in *Arabidopsis*. The floral positions are denoted by numbers, with the first flower displaying visible white petals at the inflorescence apex designated as position one. Red arrows point to the floral organ AZ. Bar equals 1 mm. **b** Heterologous expression of *LcMPK3* or *LcMPK6* under the *AtHAE* promoter in *Arabidopsis mpk3 mpk6*^*KR*^ mutants restores the floral organ abscission process, as indicated by the red arrows marking the attached floral organs. Bar equals 5 mm. **c** BCECF fluorescence from the floral organ AZ in *LcMPK3*/*6* transgenic lines and wild-type Col plants is captured. Chlorophyll autofluorescence and BCECF fluorescence are merged using confocal laser scanning microscopy (CLSM) to produce the images. Green fluorescence signifies an elevated pH. The images presented for each floral position and plant type are representative of 3 to 4 replicates. Bar equals 200 μm

To determine the cellular localization of LcMPK3 and LcMPK6, we introduced constructs containing the full-length coding sequences of *LcMPK3* and *LcMPK6* fused with green fluorescent protein (GFP) into *Nicotiana benthamiana* leaves. The results, as depicted in Fig. 2, showed that both LcMPK3- and LcMPK6-GFP signals were localized to the cell membrane and nucleus (Fig. 1c).

### LcMPK3 and LcMPK6 share conserved function in inducing floral organ abscission in *Arabidopsis*

To elucidate the potential involvement of LcMPK3 and LcMPK6 in the abscission of plant organs, we analyzed the activity patterns of their respective promoters in *Arabidopsis*. To this end, we inserted promoter fragments of *LcMPK3* or *LcMPK6* into the pCAMBIA1391 vector, which is equipped with a β-glucuronidase (GUS) reporter gene. The resulting recombinant plasmids were then introduced into *Arabidopsis* plants. In the wild-type *Arabidopsis*, the floral organs—sepals, petals, and stamens—are fully abscised at stage P8 (position 8) or P9. As shown in Fig. 2a, robust GUS activity was initially observed in the floral abscission zones of plants harboring *ProLcMPK3:GUS* at stages P5. Comparable GUS activity was also detected in plants with *ProLcMPK6:GUS*. These results indicate a specific and strong expression of *LcMPK3*/*6* in the floral organ AZ prior to abscission in *Arabidopsis.*

Furthermore, we introduced *LcMPK3* or *LcMPK6* into the *Arabidopsis mpk3 MPK6*^*KR*^ mutant, which lacks functional MPK3 and MPK6 and exhibits deficiency in floral organ abscission (Cho et al. 2008). Given the essential roles of MPK3 and MPK6 in plant development and growth, to mitigate any negative impacts from the overexpression of *LcMPK3* or *LcMPK6*, we placed their expression under the control of the *Arabidopsis HAE* promoter, which is specifically active in the floral organ AZ (Cho et al. 2008). We successfully generated 10 independent transgenic lines for both *AtHAE:LcMPK*3 and *AtHAE:LcMPK6*, each verified by quantitative reverse transcription-PCR analysis (Figure S2). After identifying homozygous transgenic lines, we observed the floral organ abscission phenotype. Notably, all *AtHAE:LcMPK3* transgenic plants in the *mpk3 MPK6*^*KR*^ background exhibited a normal floral organ abscission process, resembling that of the wild-type Col (Fig. 2b). A similar phenotype was also observed in the *AtHAE:LcMPK6* plants within the same mutant background (Fig. 2b).

Prior research has established a link between the abscission of floral organs in *Arabidopsis* and an elevation in the pH of the cytoplasm within the AZ cells, as indicated by staining with the dye 2’,7’-bis-(2-carboxyethyl)-5-(and-6)-carboxyfluorescein (BCECF)–acetoxymethyl ester (Sundaresan et al. 2015). Figure 2c illustrates that in the wild-type Col plants, distinct BCECF green fluorescence is initially detected at the abscission zone of P5 stage flowers. Conversely, in *mpk3 MPK6*^*KR*^ plants, no such BCECF green fluorescence was observed at the floral organ AZ. Intriguingly, the BCECF signals reemerged at stage 5 in the *mpk3 MPK6*^*KR*^ plants when expressing *AtHAE:LcMPK3* or *AtHAE:LcMPK6* (Fig. 2c). These observations collectively suggest that LcMPK3/6 plays a role analogous to that of *Arabidopsis* MPK3/6 in activating the abscission of floral organs.

### Silencing of *LcMPK3* or *LcMPK6* significantly suppresses the fruitlet abscission in *litchi*

**Fig. 3:**
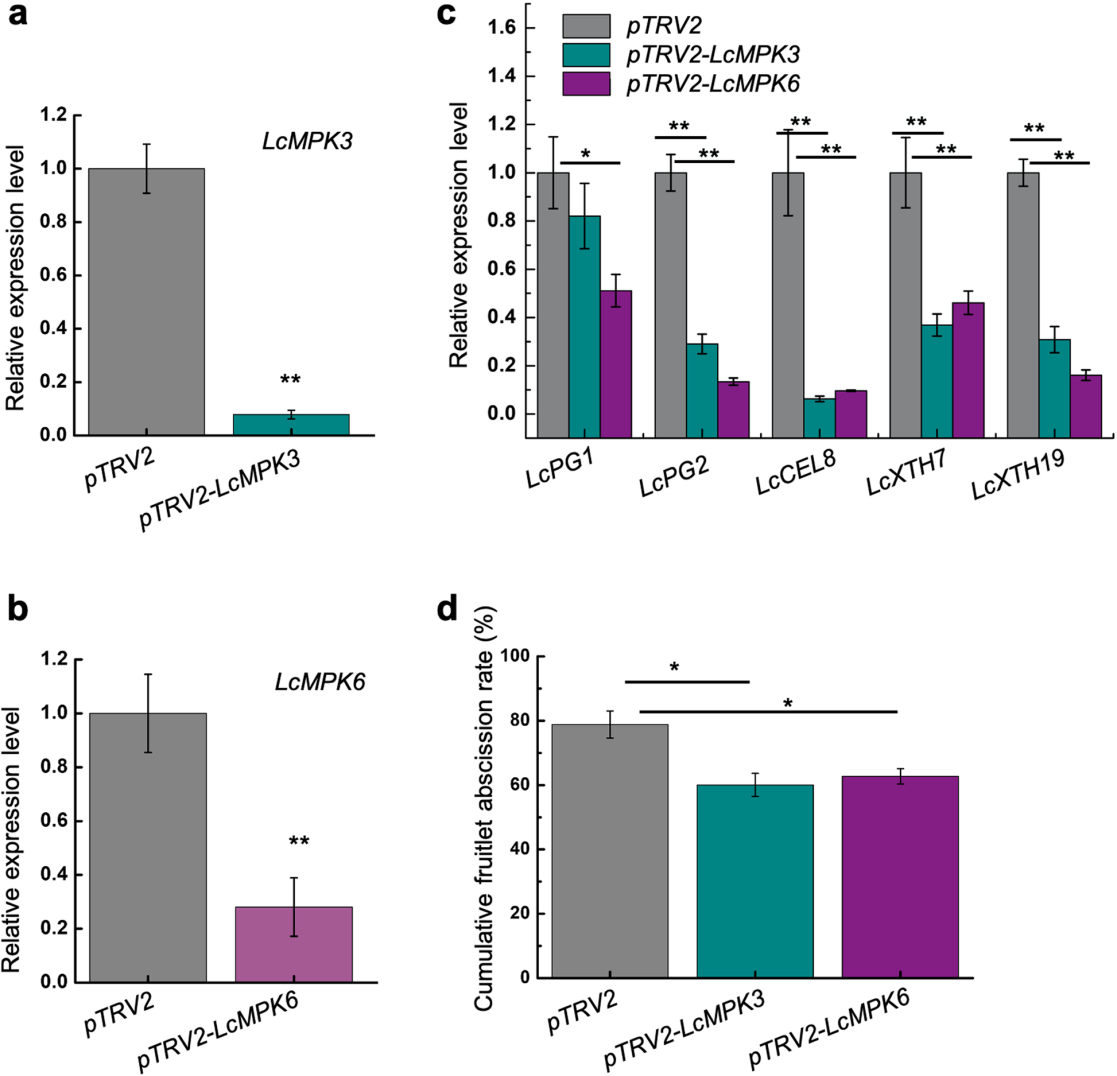
Silencing of *LcMP3* or *LcMPK6* reduces the fruitlet abscission. a Quantitative real-time PCR validated that the expression of *LcMPK3* was markedly suppressed through virus-induced gene silencing. **b** Quantitative real-time PCR validated that the expression of *LcMPK6* was markedly suppressed through virus-induced gene silencing. **c** In fruitlets with silenced *LcMPK3* or *LcMPK6*, the expression of *LcPG1*, *LcPG2*, *LcCEL8*, *LcXTH4*, and *LcXTH19* was observed to be down-regulated in the abscission zone (AZ). **d** The cumulative fruitlet abscission rate was recorded following the silencing of *LcMPK3* or *LcMPK6*. Each experiment was conducted with three biological replicates. The data are presented as the mean ± standard deviation (SD), with asterisks (*) or (**) indicating statistical significance at *P* < 0.05 or *P* < 0.01, respectively, as determined by Student’s *t*-test

To investigate the function of LcMPK3 and LcMPK6 in litchi fruitlet abscission, we suppressed *LcMPK3* or *LcMPK6* transcript levels in litchi fruitlet abscission zone (FAZ) using virus-induced gene silencing (VIGS). As shown in Fig. 3a, the expression of *LcMPK3* in the *LcMPK3*-silenced FAZ cells was reduced by approximately tenfold compared with that in control, and the expression of *LcMPK6* in the *LcMPK6*-silenced FAZ cells was reduced by approximately threefold compared with that in control (Fig. 3b). Previously, we showed that genes encoding cell wall remodeling enzymes, including LcCEL2/8, LcPG1/2, and LcXTH4/7/19, are crucial for litchi fruitlet abscission (Li et al. 2019; Ma et al. 2020; Ma et al. 2021a, b). Here, we found that *LcCEL8*, *LcPG2*, *LcXTH7*, and *LcXTH19* were significantly down-regulated in both *LcMPK3-*silenced FAZ and *LcMPK6-*silenced FAZ (Fig. 3c). Consistently, the cumulative fruitlet abscission in *LcMPK3-*silenced and *LcMPK6-*silenced fruitlets was 60.1% and 62.7%, respectively, which were significantly lower than that in control (78.9%) (Fig. 3d), suggesting that both LcMPK3 and LcMPK6 act as positive regulators in control of litchi fruitlet abscission.

### Identification of proteins interacting with LcMPK3/6

To identify potential substrates of LcMPK3 and LcMPK6 involved in litchi fruitlet abscission, we conducted a yeast two hybrid (Y2H) assay to identify proteins that associate with LcMPK3. For this purpose, a cDNA library was created from AZ samples across various stages of the abscission process, which served as the prey library (AD library). LcMPK3 was utilized to construct the bait. Following strict screening and sequencing, a total of 49 putative interacting proteins were discovered (Table 1). Subsequent analysis revealed that these candidate proteins included two components of the MAPK signaling cascade (MPK3 and MKK9), five transcriptional factors (BZR1, NAC72, KNAT3, ZFP, ERF-1), and two types of aquaporins (PIP2;5 and TIP1;1).

**Table 1:** The identification of candidate proteins interacting with LcMPK3 through Y2H screening

ID	Description	Closest Arabidopsis homolog
LITCHI024767	Mitogen-activated protein kinase 3	AT3G45640
LITCHI023007	Mitogen-activated protein kinase kinase 9	AT1G73500
LITCHI006688	BES1/BZR1 homolog protein 2S	AT3G45640
LITCHI013207	NAC domain-containing protein 72	AT4G27410
LITCHI016932	Homeobox protein knotted-1-like 3	AT5G25220
LITCHI027845	DNL-type zinc finger protein	AT3G54826
LITCHI029103	ethylene response factor	AT4G17500
LITCHI027285	Probable aquaporin PIP2-5	AT3G54820
LITCHI018676	Aquaporin TIP1-1	AT2G36830
LITCHI021068	Expansin-B3	AT4G28250
LITCHI001804	Glucan endo-1,3-beta-glucosidase 7	AT4G34480
LITCHI013896	Glucan endo-1,3-beta-glucosidase 3	AT4G26830
LITCHI027260	Probable pectate lyase 13	AT5G04310
LITCHI005118	Hypersensitive-induced response protein 1	AT5G62740
LITCHI000290	Hypersensitive-induced response protein-like protein 1	AT5G62740
LITCHI022202	Hypersensitive-induced response protein-like protein 1	AT5G62740
LITCHI000429	Ferredoxin-1	AT1G60950
LITCHI000431	Multiple organellar RNA editing factor 1	AT4G20020
LITCHI001279	Glycine-rich RNA-binding protein GRP1A	AT2G21660
LITCHI001819	Glycine-rich RNA-binding protein GRP1A	AT2G21660
LITCHI001928	Fructose-bisphosphate aldolase 2	AT4G38970
LITCHI002792	Osmotin-like protein OSM34	AT4G11650
LITCHI002793	Osmotin-like protein OSM34	AT4G11650
LITCHI030236	Osmotin-like protein OSM34	AT4G11650
LITCHI011513	Peroxidase 12	AT1G71695
LITCHI024186	Peroxidase 15	AT5G06730
LITCHI023452	Catalase	AT3G47490
LITCHI004474	Methyltransferase-like protein 13	AT3G60910
LITCHI004685	Nuclear pore complex protein NUP54	AT1G24310
LITCHI030959	Nuclear pore complex protein NUP96	AT1G80680
LITCHI004991	Protein RICE SALT SENSITIVE 3	AT1G60060
LITCHI005730	Beta-glucuronosyltransferase GlcAT14A	AT1G71070
LITCHI009151	Thaumatin-like protein	AT4G11650
LITCHI009301	Thaumatin-like protein	AT4G11650
LITCHI009352	Ferredoxin-dependent glutamate synthase	AT5G52390
LITCHI013320	Probable NAD(P)H dehydrogenase (quinone) FQR1-like 1	AT4G27270
LITCHI016817	COP9 signalosome complex subunit 5a	AT1G22920
LITCHI017243	piRNA biogenesis protein EXD1	AT2G25910
LITCHI018791	Fructose-bisphosphate aldolase 6	AT2G36460
LITCHI019379	UDP-arabinopyranose mutase 1	AT3G02230
LITCHI020520	Formate dehydrogenase	AT5G14780
LITCHI021839	Methionine gamma-lyase	AT1G64660
LITCHI021841	Methionine gamma-lyase	AT1G64660
LITCHI021848	Methionine gamma-lyase	AT1G64660
LITCHI025219	SWR1 complex subunit 6	AT5G37055
LITCHI025223	SWR1 complex subunit 6	AT5G37055
LITCHI028757	Endoplasmic reticulum oxidoreductin-2	AT2G38960
LITCHI029807	Serine/threonine-protein kinase D6PKL2	AT5G47750
LITCHI031097	CBL-interacting serine/threonine-protein kinase 11	AT2G30360

**Fig. 4:**
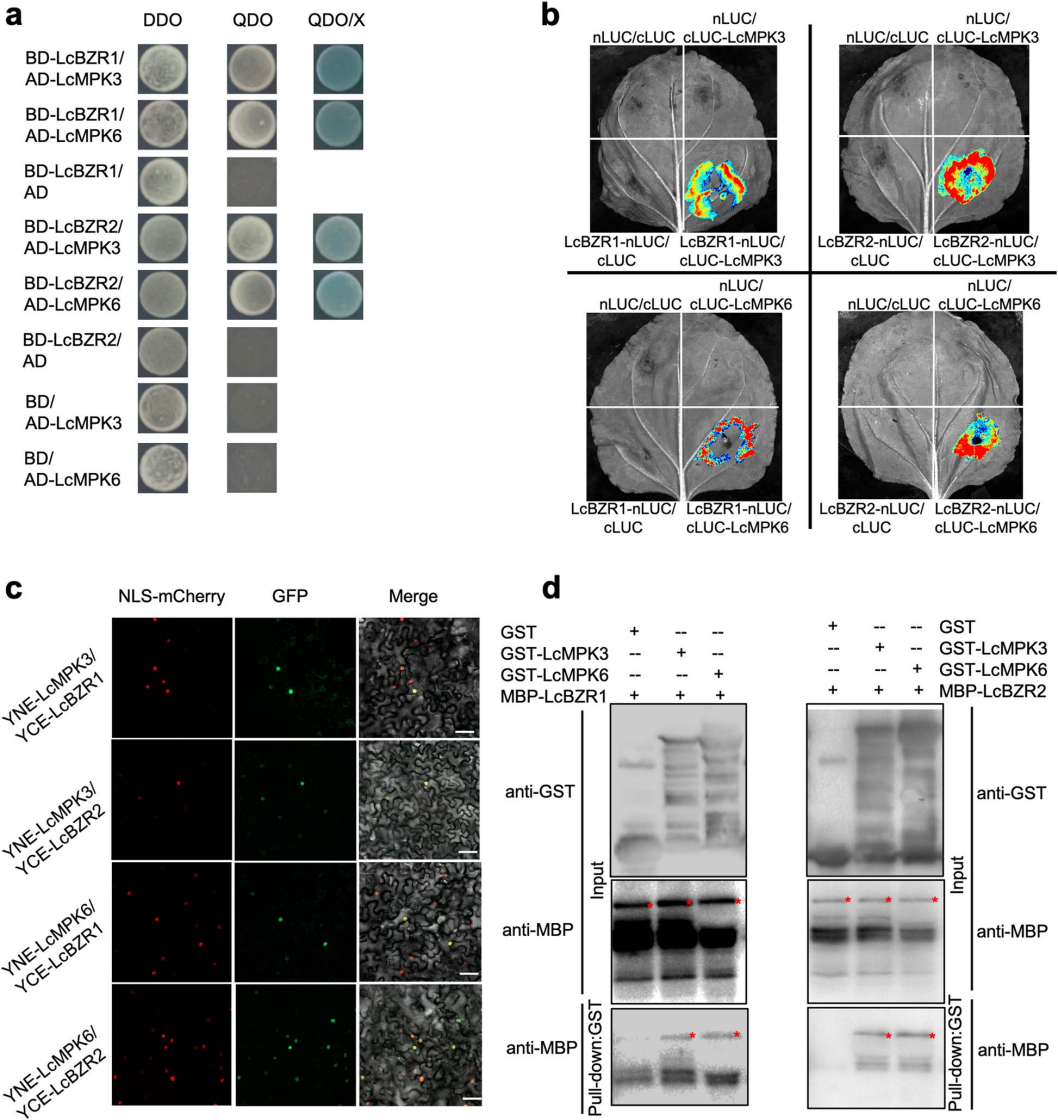
LcMPK3/6 interact with LcBZR1/2 in vivo and in vitro. **a** The interaction between LcMPK3/6 and LcBZR1/2 was confirmed using a yeast two-hybrid (Y2H) assay. DDO refers to synthetic defined (SD) medium lacking tryptophan (Trp) and leucine (Leu), while QDO is SD medium lacking Trp, Leu, histidine (His), and adenine (Ade). QDO/X denotes QDO medium supplemented with X-α-galactosidase. **b** The interaction was further demonstrated in *Nicotiana benthamiana* leaves using a split-luciferase assay. LcBZR1-nLUC (or LcBZR2-nLUC) was co-expressed with cLUC-LcMPK3 (or cLUC-LcMPK6), with LcBZR1/2-nLUC/cLUC, nLUC/cLUC-LcMPK3/6, and nLUC/cLUC serving as negative controls. Luciferase activity was recorded using a CCD camera, and representative images of the leaves 72 h post-infiltration are displayed. **c** Bimolecular fluorescence complementation (BiFC) assays in tobacco leaves confirmed the interaction between LcMPK3/6 and LcBZR1/2. YCE-LcBZR1 (or YCE-LcBZR2) was co-expressed with YNE-LcMPK3 (or YNE-LcMPK6), and the mCherry driven by the nuclear localization signal (NLS-mCherry) and GFP fluorescence were visualized under a confocal laser scanning microscope. Scale bars represent 100 μm. **d** Pull-down assays further validated the interaction between LcMPK3/6 and LcBZR1/2. Recombinant GST-LcMPK3 (or GST-LcMPK6) immobilized on glutathione Sepharose beads was incubated with MBP-LcBZR1 (or MBP-LcBZR2). The eluted proteins were detected by immunoblotting with anti-GST and anti-MBP antibodies, with red stars indicating the positions of MBP-LcBZR1 or MBP-LcBZR2

Given the established role of LcBZR1/2 in inhibiting fruitlet abscission in litchi (Ma et al. 2021a, b), we chose to verify the interaction between LcBZR1 and LcMPK3. Initially, we conducted the Y2H assay once more. The Y2H gold yeast cells, which were co-transformed with AD-LcMPK3 (the full-length LcMPK3 fused to pGADT7) and BD-LcBZR1 (the full-length LcBZR1 fused to pGBKT7), were capable of growth on the selective medium QDO (a synthetic medium devoid of tryptophan, leucine, histidine, and adenine), and turned blue in the presence of x-a-gal (Fig. 4a). Similarly, cells co-transformed with AD-LcBZR1 and BD-LcMPK3 were also able to grow on QDO medium. In contrast, cells co-transformed with BD-LcBZR1 and an empty AD vector or with AD-LcMPK3 and an empty BD vector did not grow on QDO medium (Fig. 4a). This outcome confirms that LcMPK3 can directly interact with LcBZR1 within the yeast system. Additionally, our findings revealed that LcMPK3 can also interact with LcBZR2, and that LcMPK6 can interact with both LcBZR1 and LcBZR2 in the yeast system (Fig. 4a).

Subsequently, we conducted split luciferase complementation imaging (LCI) assays to further investigate the interactions. In these assays, we co-expressed LcBZR1-nLUC (or LcBZR2-nLUC) with LcMPK3-cLUC (or LcMPK6-cLUC) transiently in *Nicotiana benthamiana* leaves. We observed robust luciferase activity in the leaves co-expressing these constructs, which was absent in the negative control samples (Fig. 4b), confirming the in vivo interaction between LcMPK3/6 and LcBZR1/2 within the plant.

Moving forward, we performed bimolecular fluorescence complementation (BiFC) assays in *Nicotiana benthamiana* leaves. We transiently co-expressed LcMPK3 or LcMPK6, each tagged with pSPYNE (the N-terminal fragment of split YFP), with LcBZR1 or LcBZR2, each tagged with pSPYCE (the C-terminal fragment of split YFP). As shown in Fig. 4c, a strong YFP fluorescence signal was observed in the nuclei of tobacco cells co-expressing either YNE-LcMPK3 or YNE-LcMPK6 with YCE- LcBZR1 or YCE- LcBZR2, whereas no YFP signal was detected in the negative control cells (Figure S3). These BiFC results not only confirmed the in vivo interactions between LcMPK3/6 and LcBZR1/2 but also revealed the specific nuclear localization of the interacting proteins, aligning with nuclear localization of LcMPK3/6 and LcBZR1/2 (Fig. 1c) (Ma et al. 2021a, b).

To finally confirm the in vitro interactions between LcMPK3/6 and LcBZR1/2, we employed a pull-down assay. We allowed recombinant glutathione S-transferase (GST)-fused LcMPK3/6 (GST-LcMPK3 or GST-LcMPK6) attached to glutathione sepharose beads to interact with maltose-binding protein (MBP)-fused LcBZR1/2 (MBP-LcBZR1 or MBP-LcBZR2). The resulting complexes were then analyzed using immunoblotting. As shown in Fig. 4d, GST-LcMPK3 or GST-LcMPK6, in contrast to GST alone, were capable of directly interacting with MBP-LcBZR1 or MBP-LcBZR2. Taken together, we conclude that LcMPK3/6 forms a complex with LcBZR1/2 both in vitro and in vivo.

### LcMPK3/6 phosphorylate LcBZR1/2

**Fig. 5:**
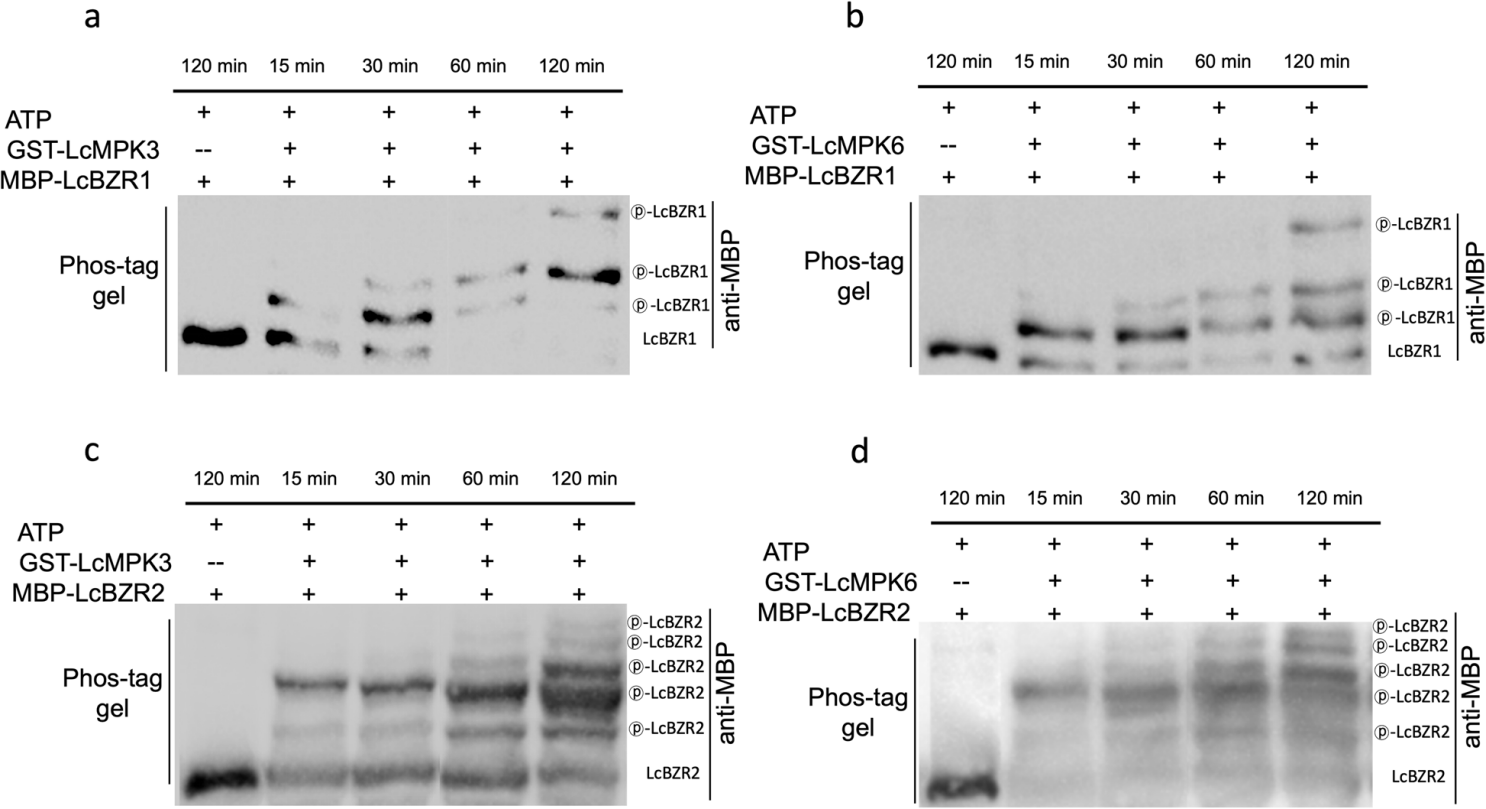
In vivo phosphorylation assay of LcBZR1/2 phosphorylated by LcMPK3/6. **a** An in vivo phosphorylation assay of the phosphorylation of LcBZR1 by LcMPK3 using Phos-tag. Recombinant MBP-LcBZR1 and GST-LcMPK3 were incubated in kinase buffer, and phosphorylation was detected using Phos-tag SDS-PAGE. **b** An in vivo phosphorylation assay of the phosphorylation of LcBZR1 by LcMPK6 using Phos-tag. **c** An in vivo phosphorylation assay of the phosphorylation of LcBZR2 by LcMPK3 using Phos-tag. **d** An in vivo phosphorylation assay of the phosphorylation of LcBZR2 by LcMPK6 using Phos-tag. Incubation times varied (15, 30, 60, and 120 min), and phosphorylation was detected by immunoblotting with an anti-MBP antibody. The circled “p” denotes the phosphorylated state

The interaction observed between LcMPK3/6 and LcBZR1/2 proteins led us to explore the possibility of LcBZR1/2 being phosphorylated by LcMPK3/6. We conducted an in vitro phosphorylation assay using Phos-tag SDS–PAGE. Figure 5 illustrates that in the absence of LcMPK3 or LcMPK6, LcBZR1 displayed a single band. However, with LcMPK3 or LcMPK6 present, there was a clear indication of LcBZR1 phosphorylation, as seen by the mobility shifts in the Phos-tag gel. Intriguingly, as the incubation period increased, the number of bands increased from one to three, suggesting that LcMPK3 phosphorylated multiple sites on LcBZR1 (Fig. 5a, b). Notably, multiple shift bands were also observed for the phosphorylation of LcBZR1 and LcBZR2 by both LcMPK3 and LcMPK6 (Fig. 5c, d).

**Fig. 6:**
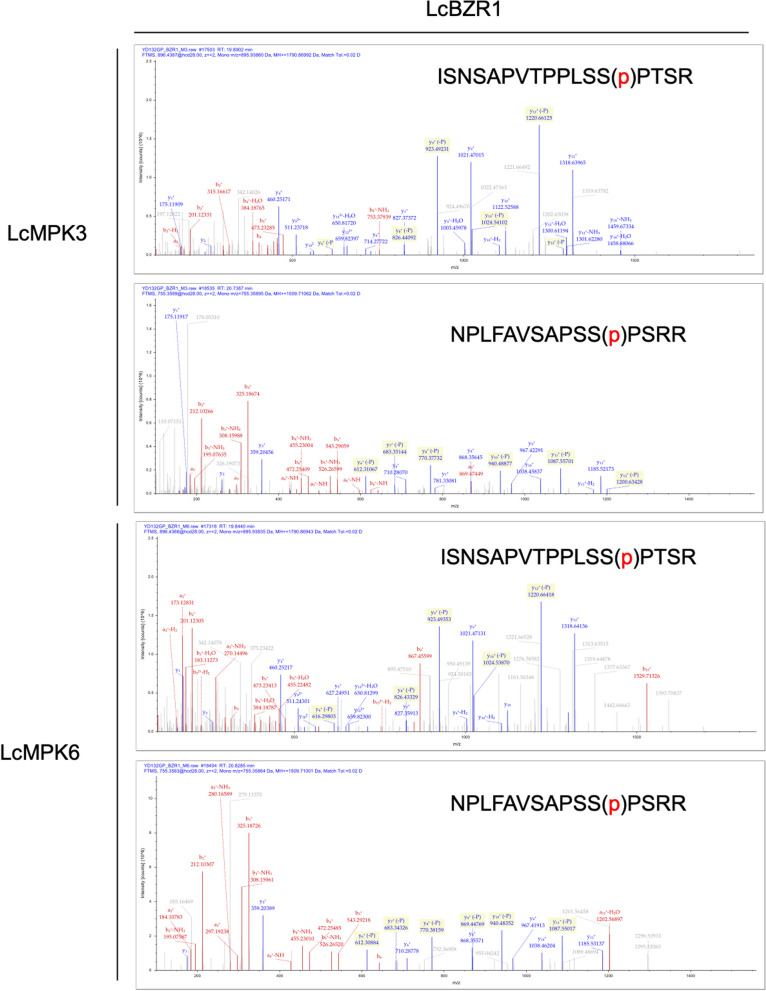
Identification of the Phosphorylation sites on LcBZR1 by LcMAPK3/6 using liquid chromatography-tandem mass spectrometry (LC–MS/MS). The mass spectra of the phosphopeptides are depicted, with b-ions and y-ions alongside their respective peptide sequences. The phosphorylated serine (S) residues are indicated with a (p) symbol

To pinpoint the exact phosphorylation sites within LcBZR1 and LcBZR2, we treated these recombinant proteins with LcMPK3 or LcMPK6 to trigger phosphorylation events. The phosphorylated proteins were subsequently analyzed by liquid chromatography tandem mass spectrometry (LC–MS/MS). Our analysis revealed two high-confidence phosphorylation sites (Ser-168 and Ser-203) for LcBZR1 when in the presence of either LcMPK3 or LcMPK6 (Fig. 6). For LcBZR2, we identified five phosphorylation sites (Ser-85, Ser-121, Thr-164, Ser-196, and Ser-242) with LcMPK3 (Figure S4) and another set of five sites (Ser-121, Ser-143, Ser-196, Ser-237, and Ser-242) with LcMPK6 (Figure S5) (Table 2).

**Table 2:** The Phosphorylation sites of LcBZR1/2 by LcMPK3/6

Kinase	Substrate	Phosphorylation peptide	Phosphorylation site
LcMPK3	LcBZR1	ISNSAPVTPPLSS(p)PTSR	S168
		NPLFAVSAPSS(p)PSRR	S203
LcMPK6	LcBZR1	ISNSAPVTPPLSS(p)PTSR	S168
		NPLFAVSAPSS(p)PSRR	S203
LcMPK3	LcBZR2	GMKPS(p)PIDIASSSTR	S85
		ITPYSSQNPSPLSSAFASPAPSYPTS(p)PTR	S121
		ISNSAPVTPPLSSPT(p)KHPK	T164
		ESMSTFNYPFYAISAPAS(p)PIHR	S196
		YAQPSASGMPTS(p)PTFNLVR	S242
LcMPK6	LcBZR2	ITPYSSQNPSPLSSAFASPAPSYPTS(p)PTR	S121
		NAIPS(p)SLPPLR	S143
		ESMSTFNYPFYAISAPAS(p)PIHR	S196
		YAQPSAS(p)GMPTSPTFNLVRPVAPQAFANDAMK	S237
		YAQPSASGMPTS(p)PTFNLVR	S242

## Discussion

Plant organ abscission is a highly regulated process, with the MAPK cascades identified as key regulatory components (Cho et al. 2008). However, the specific protein substrates phosphorylated by MAPK cascades during abscission and the conservation of their role across plant species are not yet established. In this study, we demonstrate that LcMPK3/6 serve as positive regulators in the fruit abscission of litchi. Utilizing the Y2H system, we identified potential substrates of LcMPK3/6 and confirmed their interactions with the transcription factors LcBZR1/2 both in vivo and in vitro. These findings provide fundamental information to explore the mechanisms of abscission mediated by MPK3/6.

The MAPK cascade, specifically the MKK4/5-MPK3/6 module, has been identified as a central regulatory component in *Arabidopsis* floral organ abscission (Cho et al. 2008). Mutations disrupting both MPK3 and MPK6, which lie downstream of MKK4 and MKK5, result in impaired floral organ abscission (Cho et al. 2008). However, the conservation of this cascade in other plant species’ organ abscission processes is not yet clear. Our research showed that the ectopic expression of *LcMPK3* or *LcMPK6* in the floral organ abscission zone of *Arabidopsis mpk3 mpk6*^*KR*^ mutants can restore the abscission process (Fig. 2), indicating a conserved function with their *Arabidopsis* homologs. Additionally, the positive role of LcMPK3 and LcMPK6 in abscission was confirmed in litchi by silencing their expression, which significantly reduced fruitlet abscission rates (Fig. 3). These findings provide evidence for the conserved role of MPK3/6 in the regulation of organ abscission across different plant species. It is of note that *Arabidopsis* MPK3 and MPK6 function redundantly in regulating the floral organ abscission, while whether it is the knockdown of *LcMPK3* or the knockdown of *LcMPK6* via VIGS, both can significantly reduce the litchi fruitlet abscission, indicating that the role of LcMPK3 and LcMPK6 in litchi fruitlet abscission is synergistic.

Posttranslational phosphorylation is a critical regulatory mechanism for proteins, influencing their activity, localization, interactions, and stability (Kwon et al. 2006). The phosphorylation of transcription factors, in particular, has garnered attention due to their pivotal roles in signal transduction. For instance, the phosphorylation of MdNAC72 by MdMAPK3 facilitates its degradation, hastening apple fruit softening (Wei et al. 2023). In citrus, CsMYC2 phosphorylation by CsMPK6 reduces its stability and DNA-binding capacity, promoting fruit coloration (Yue et al. 2023). Similarly, in strawberry, FvRIF is phosphorylated by the FvMKK4–FvMAPK6 module, enhancing its transcriptional activation and fruit ripening (Li et al. 2023). Our study identified five transcription factors—BZR1, NAC72, KNAT3, ZFP, and ERF-1—that interact with LcMPK3 in yeast. Notably, LcBZR1/2, known to suppress litchi fruitlet abscission by downregulating ethylene biosynthesis genes (Ma et al. 2021a, b), were confirmed to interact with LcMPK3/6 both in vitro and in vivo (Fig. 4). It is well known that posttranslational phosphorylation modulates the stability, nuclear localization, and DNA-binding activity of BZR transcription factors. In the absence of brassinosteroids, active GSK3-like kinase BRASSINOSTEROID INSENSITIVE2 (BIN2) phosphorylates BZR1 and BZR2, inhibiting their DNA-binding and sequestering them in the cytoplasm via 14–3-3 proteins (He et al. 2002; Wang et al. 2002; Yin et al. 2002; Vert and Chory 2006; Bai et al. 2007; Gampala et al. 2007). Conversely, high BR levels lead to dephosphorylation by PROTEIN PHOSPHATASE 2A (PP2A), allowing BZR1 and BZR2 to enter the nucleus and regulate gene expression (He et al. 2005; Yin et al. 2005; Sun et al. 2010; Tang et al. 2011; Yu et al. 2011). During banana fruit ripening, it has been reported that MaMPK14 can phosphorylate MaBZR1/2 and enhance their transcriptional inhibitory ability on genes related to cell wall remodeling (Shan et al. 2020). In our study, we demonstrated that LcBZR1/2 were phosphorylated by LcMPK3/6, and multiple phosphorylation sites were identified (Fig. 5, S4, S5). Given that LcBZR1/2 can repress ethylene biosynthesis genes during litchi fruitlet abscission, it is intriguing to explore whether LcMPK3/6-mediated phosphorylation could modulate the transcriptional activity of LcBZR1/2 on these targets in future studies.

In *Arabidopsis*, the floral organ abscission signaling pathway involves the MAPK cascade phosphorylating the MADS domain transcription factor AGAMOUS-LIKE 15 (AGL15). This phosphorylation event derepresses the expression of *HAE*, thereby negatively regulating floral organ abscission (Patharkar and Walker 2015). While AGL15 is the only known potential substrate of the MKK4/5-MPK3/6 module, direct interaction between AGL15 and the module has not been experimentally verified. In our study, we utilized Y2H system to identify proteins interacting with LcMPK3 (Table 1). Recent findings suggest that tonoplast intrinsic proteins (TIPs), part of the aquaporin family, play a crucial role in tomato pedicel abscission. The knockout of *SlTIP1;1* leads to delayed abscission, while overexpression accelerates the process. Further investigation revealed that SlTIP1;1 influences pedicel abscission by increasing the levels of hydrogen peroxide in the cytoplasm and the osmotic water permeability (P_f_), which is essential for increasing turgor pressure and in turn provides the necessary force for the separation of the abscission zone cells (Wang et al. 2021). Our research identified two aquaporin proteins, PIP2;5 and TIP1;1, as interactors with LcMPK3 (Table 1). Aquaporins, which regulate water transport, are subject to posttranslational modifications such as phosphorylation, which can induce a conformational change, switching the aquaporin between ‘closed’ and ‘open’ states, and thus modulate water permeability (Tornroth-Horsefield et al. 2006; Nyblom et al. 2009; Maurel et al. 2015). For instance, the aquaporin PIP2;2 is phosphorylated and activated by calcium-dependent protein kinase 16, governing the reversible opening of *Gentiana scabra* flowers (Nemoto et al. 2022). Thus, our discovery raises the intriguing possibility that a LcMPK3/6-LcPIP2;5/LcTIP1;1 module may be implicated in litchi fruitlet abscission via regulating the ROS production and cell turgor pressure, warranting further investigation.

Additionally, transcription factors such as SlERF52 in tomato, RhERF1/4 in rose, KNATs across *Arabidopsis*, tomato, and litchi, and ZFP2 in *Arabidopsis* have been implicated in the abscission process (Shi et al. 2011; Nakano et al. 2014; Ma et al. 2015; Gao et al. 2019; Zhao et al. 2020; Wang et al. 2021). However, the potential phosphorylation of these factors during abscission is an area that requires further investigation. Although our study identified interactions between NAC72, KNAT3, ZFP, and ERF-1 with LcMPK3 in yeast, these interactions require in vivo validation further. Importantly, determining whether these transcription factors are phosphorylated by LcMPK3/6 and their subsequent involvement in litchi fruitlet abscission is a critical next step in future research.

**Fig. 7:**
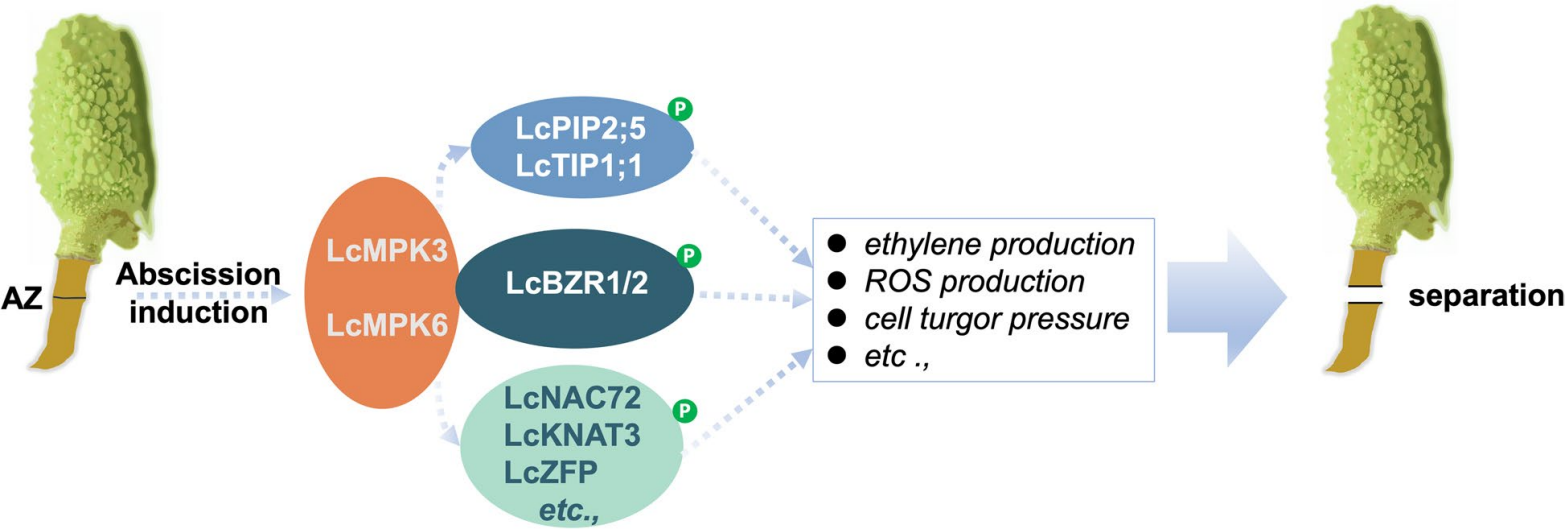
Proposed model of LcMPK3/6-mediated signaling pathway in litchi fruit abscission. Upon abscission induction, core downstream components in the MAPK cascade, LcMPK3 and LcMPK6, are activated. LcMPK3/6 facilitate fruit abscission, likely through interacting and phosphorylating various proteins such as LcBZR1/2, LcPIP2;5, LcTIP1;1, LcNAC72, among others, to enhance ethylene production, ROS production, and cell turgor pressure, etc. These actions, in turn, promote cell separation in the abscission zone

In conclusion, we propose that the MAPK cascade in the AZ is activated upon litchi fruit abscission induction by internal or external cues. The core components LcMPK3 and LcMPK6 work together to regulate abscission, likely by phosphorylating various proteins such as LcBZR1/2, LcPIP2;5, LcTIP1;1, etc., to increase ethylene production, ROS production and cell turgor pressure, etc., thereby promoting cell separation in the AZ and ultimately leading to fruit abscission (Fig. 7).

## Materials and methods

### Plant materials and treatment

The ‘Nuomici’ litchi trees (*Litchi chinensis* Sonn.) selected from the China National Litchi Germplasm Resource Nursery, Institute of Fruit Tree Research, Guangdong Academy of Agricultural Sciences, were utilized for the virus-induced gene silencing (VIGS) experiments. Concurrently, all *Arabidopsis thaliana* accessions of the Columbia (Col-0) ecotype, employed in this study, were cultivated under a regimen of extended photoperiods, specifically at a temperature of 22 °C with a light/dark cycle of 16 h illumination and 8 h darkness at an intensity of 100 μmol m^−2^ s^−1^.

### Sequence alignment, isolation of genes, and qRT-PCR

The litchi MAPK gene sequences were extracted from the litchi genome dataset using TBtools (Chen et al. 2023) (http://121.37.229.61:82/), while the *Arabidopsis* MAPK genes were sourced from the TAIR database (https://www.arabidopsis.org/). Multiple sequence alignments were conducted using ClustalW and GeneDoc. MEGA 11 was utilized to construct a phylogenetic tree, employing the Poisson correction model and the neighbor-joining (NJ) method.

Total RNA was isolated from the abscission zone (AZ) tissues of litchi fruitlets and the floral organ AZ of Arabidopsis flowers (P3-P8, counted from the first flower with visible white petals) using 1 mL of Trizol reagent (Invitrogen). The first strand cDNA synthesis was performed using 2 μg of total RNA, following the manufacturer’s protocol for the TransScript One-Step gDNA Removal and cDNA Synthesis SuperMix Kit (TransGen, Beijing). The experiment was conducted in triplicate for both litchi and *Arabidopsis*, with *EF-1a* and *Ubiquitin 10* (*UBQ10*) serving as internal controls, respectively (Zhong et al. 2011). All PCR reactions were normalized to the reference gene using a Ct value. The relative expression levels of the target genes were quantified using the 2^−ddCt^ method (Livak and Schmittgen 2001).

### Subcellular localization assay

Full-length cDNAs of *LcMPK3* and *LcMPK6* was inserted into the pC18-GFP vector, and the resulting fusion plasmids were transformed into *Agrobacterium tumefaciens* strain GV3101-psoup-p19. This *Agrobacterium*, with an optical density (OD_600_) of approximately 0.2, was used to infect tobacco (*Nicotiana benthamiana*) leaves individually. After approximately 48 h, the fluorescence signals were visualized using a confocal laser scanning microscope (ZEISS LSM 7 DUO).

### Histochemical GUS assays

The 2 kb promoter region of *LcMPK3* or *LcMPK6* was cloned into the pCAMBIA1391-GUS vector. These recombinant plasmids were introduced into *Agrobacterium tumefaciens* strain GV3101, which was subsequently used to transform *Arabidopsis* Col-0 plants via the floral dip method (Clough and Bent 1998). GUS staining and tissue decolorization were performed according to the protocol outlined in a prior study (Ma et al. 2019).

### *Arabidopsis* genetic transformation

To achieve overexpression of *LcMPK3* or *LcMPK6* in *Arabidopsis mpk3 mpk6*^*KR*^ double mutants, the full-length coding sequence each was cloned into the pCAMBIA1302 vector, which features the 35S promoter replaced by the *Arabidopsis AtHAE* promoter. The modified plasmids were introduced into *Agrobacterium tumefaciens* strain GV3101. Transformation of the *mpk3 mpk6*^*KR*^ mutants was performed using the floral dip method (Clough and Bent 1998). The presence of transgenic plants was verified through quantitative PCR (qPCR) analysis.

### BCECF assay

For the BCECF fluorescence assays, flowers from transgenic *Arabidopsis* plants expressing *AtHAE:LcMPK3* or *AtHAE:LcMPK6* were incubated in a 10 µM BCECF-AM (B1150, Invitrogen) solution in darkness for 20 min. Afterward, any unbound BCECF-AM was eliminated by rinsing with phosphate-buffered saline (PBS, pH 7.4). Fluorescence images were captured using a ZEISS LSM 7 DUO confocal laser scanning microscope, with excitation and emission settings at 470 nm and 525 nm, respectively.

### Virus induced gene silencing (VIGS)

The VIGS assay was conducted following a previous protocol (Zhang et al. 2018). In brief, a 300 bp fragment of *LcMPK3* (921–1221) or *LcMPK6* (1–300) was cloned into the pTRV2 vector to create the silencing constructs, which were then transformed into *Agrobacterium tumefaciens* strain GV3101. For the transformation, an equal mixture of cultures containing pTRV RNA1 (pTRV1) and pTRV2 constructs was used (Ratcliff et al. 2001), with a bacterial optical density (OD_600_) of approximately 1.0. The flowering panicles of the ‘Nuomici’ litchi variety were dipped into the bacterial culture for 60 s. Approximately forty panicles from different locations on a tree were chosen, with twenty designated for VIGS treatment and the remainder serving as controls. The experiment was replicated three times, with each replication involving a separate tree. The Cumulative Fruitlet Abscission Rate (CFAR) was calculated by determining the total percentage of fruitlet abscission. The CFAR was assessed 21 days post-silencing.

### Yeast two hybrid assay

The complete coding sequence of *LcBZR1* or *LcBZR2* was inserted into the pGBKT7 vector, resulting in the constructs BD-LcBZR1 or BD-LcBZR2. Similarly, the full-length coding sequence of *LcMPK3* or *LcMPK6* was cloned into the pGADT7 vector to create AD-LcMPK3 or AD-LcMPK6. These complementary pairs of plasmids were co-transformed into Y2H Gold yeast cells. The yeast strains were cultured at 30 °C on a selective medium lacking leucine, tryptophan, histidine, and adenine. The potential protein–protein interactions were assessed based on the yeast growth and α-galactosidase activity.

### LCI assay

The genes *LcBZR1* or *LcBZR2* was cloned in-frame and positioned upstream of the N-terminal half of the firefly luciferase (LUC) sequence in the pCAMBIA1300-nLUC vector. Concurrently, *LcMPK3* or *LcMPK6* was cloned in-frame and placed downstream of the C-terminal half of the luciferase in the pCAMBIA1300-cLUC vector. These vectors, along with the empty controls, were transformed into *Agrobacterium tumefaciens* strain GV3101 and transiently expressed in *Nicotiana benthamiana* leaves, following the method described previously (Chen et al. 2008). After 72 h of growth, the leaves were treated with a 1 mM D-luciferin solution in ddH_2_O with 0.01% (v/v) Triton X-100 and incubated in darkness for 5 min. Luminescence was then imaged using a chemiluminescence imager equipped with a cooled charge-coupled device (CCD) camera (Bio-Rad).

### BiFC assay

The full-length coding sequence for *LcBZR1* or *LcBZR2* was subcloned into the pUC-pSPYCE vector, and similarly, the full-length coding sequence for *LcMPK3* or *LcMPK6* was subcloned into the pUC-pSPYNE vector (Walter et al. 2004). These pairs of plasmids were then transformed into *Agrobacterium tumefaciens* strain GV3101 and co-injected into the young leaves of *Nicotiana benthamiana*. NLS-mCherry served as a nuclear marker (Wu et al. 2021). Two days post-inoculation, the GFP (excitation/emission: 470 nm/525 nm) and mCherry (excitation/emission: 550 nm/628 nm) signals were visualized using a laser confocal microscope (LSM 7 DUO; Zeiss).

### Pull-down assay

For the production of MBP-LcBZR1 or MBP-LcBZR2 and GST-LcMPK3 or GST-LcMPK6 recombinant proteins, the coding sequence of *LcBZR1* or *LcBZR2* was amplified and cloned into the pMAL-c2X-MBP vector, while the coding sequence of *LcMPK3* or *LcMPK6* was cloned into the pGEX-4 T-3 vector. These constructs were then transformed into *E. coli Rosetta* DE3 cells (TransGen Biotech, China). To purify the soluble recombinant MBP-LcBZR1 or MBP-LcBZR2 proteins, the recombinant MBP was isolated via affinity chromatography using amylose resin. Conversely, GST-LcMPK3 or GST-LcMPK6 proteins were purified using glutathione Sepharose beads. The protein mixtures were captured with a glutathione purification kit (Thermo Fisher Scientific). The eluted proteins were identified using anti-GST (Abcam, cat. no. ab9058) and anti-MBP (Abcam, cat. no. ab9084) antibodies.

### In vitro phosphorylation assay

In vitro phosphorylation assays were conducted following the established method (Li et al. 2023). The reactions contained 5 μg of recombinant substrate protein (MBP-LcBZR1 or MBP-LcBZR2) and 10 μg of kinase (GST-LcMPK3 or GST-LcMPK6) in a 100 μL kinase buffer (25 mM Tris–HCl pH 7.5, 10 mM MgCl2, 1 mM CaCl2, 10 mM ATP, and 1 mM DTT) and were incubated at 30 °C for various durations (15, 30, 60, and 120 min). The phosphorylated proteins were resolved on a 10% SDS-PAGE gel incorporating 50 μM Phos-tag and 100 μM MnCl2. Following electroblotting onto a polyvinylidene fluoride (PVDF) membrane, MBP-LcBZR1 or MBP-LcBZR2 were detected using an anti-MBP antibody (Abcam, cat. no. ab9084).

### Phosphorylation site identification

The phosphorylation sites were characterized using liquid chromatography-tandem mass spectrometry (LC–MS/MS). Samples, which included phosphorylated proteins extracted from SDS-PAGE gels, were processed through reduction, alkylation, and enzymatic digestion with trypsin and chymotrypsin. The resulting tryptic peptides were resuspended in 0.1% formic acid (solvent A) and loaded onto a homemade reversed-phase analytical column (15 cm × 75 μm i.d.). The chromatographic gradient increased solvent B (0.1% formic acid in 98% acetonitrile) from 6 to 23% over 16 min, to 35% over 8 min, and then ramped up to 80% within 3 min, holding at this level for the final 3 min, all at a flow rate of 400 nl/min on an EASY-nLC 1000 UPLC system.

Peptides were introduced via a nanospray ion source into a Q Exactive™ Plus mass spectrometer (Thermo) connected online to the UPLC. The electrospray voltage was set at 2.0 kV, with a full scan m/z range of 350 to 1800 in the Orbitrap at a resolution of 70,000. Subsequently, peptides were selected for MS/MS with a normalized collision energy (NCE) of 28, and the resulting fragments were detected at a resolution of 17,500. Data acquisition employed a data-dependent strategy, alternating one MS scan with 20 MS/MS scans, each with a 15-s dynamic exclusion window. The automatic gain control (AGC) target was set to 5 × 10^4^.

The acquired MS/MS data were analyzed using Proteome Discoverer 1.3. Tandem mass spectra were searched against the litchi protein database (http://121.37.229.61:82/). Trypsin/P was designated as the cleavage enzyme, allowing for up to two missed cleavages. Mass tolerances were set at 10 ppm for precursor ions and 0.02 Da for fragmentions. Carbamidomethylation of cysteine was defined as a fixed modification, while oxidation of methionine was considered a variable modification. Peptide identification was filtered for high confidence, with a minimum ion score set at > 20.

### Supplementary Information

Additional file 1. Figure S1. The chromosomal distribution of litchi MPKs.

Additional file 2. Figure S2. The relative expression level of *LcMPK3* in the floral organ AZ of *Arabidopsis mpk3 mpk6*^*KR*^ mutants expressing *AtHAE:LcMPK3 *(a), and the relative expression level of *LcMPK6* in the floral organ AZ of *Arabidopsis mpk3 mpk6*^*KR*^ mutants expressing *AtHAE:LcMPK6 *(b).

Additional file 3. Figure S3. Negative controls of BiFC assays. YCE-LcBZR1 (or YCE-LcBZR2) was co-expressed with YNE, and YNE-LcMPK3 (or YNE-LcMPK6) was co-expressed with YCE. The nuclear localization signal (NLS-mCherry) and GFP fluorescence were captured using a confocal laser scanning microscope. Scale bars indicating 100 μm are shown.

Additional file 4. Figure S4. Identification of LcBZR2 sites phosphorylated by LcMAPK3 via LC-MS/MS. The mass spectrum of peptide with phosphorylation sites is shown. The b-ions and y-ions and the corresponding peptide sequence are presented, with phosphorylated serine (S) residue or threonine (T) marked by (p).

Additional file 5. Figure S5. Identification of LcBZR2 sites phosphorylated by LcMAPK6 via LC-MS/MS. The mass spectrum of peptide with phosphorylation sites is shown. The b-ions and y-ions and the corresponding peptide sequence are presented, with phosphorylated serine (S) residue marked by (p).

Additional file 6. Table S1. The primers used in this study.

## Data Availability

The data underlying this article are available in the article and in its online supplementary material.

## Abbreviations

MAPK: Mitogen-activated protein kinase
MKK/MEK/MAPKK: MAPK kinase
MKKK/MEKK: MAPKK kinase
MPK3: Mitogen-activated protein kinase 3
MPK6: Mitogen-activated protein kinase 6
IDA: Inflorescence deficient in abscission
HAE: HAESA
HSL2: HAESA-LIKE2
BP: BREVIPEDICELLUS
AGL15: AGAMOUS-like 15
EIN3: ETHYLENE INSENSITIVE 3
ERF: Ethylene response factor
NAC: NAM、ATAF1/2、CUC1/2
RIF: Ripening inducing factor
MYC2: A basic helix-loop-helix transcription factor
KNAT: A KNOTTED-LIKE FROM ARABIDOPSIS THALIANA
ZFP: Zinc finger protein
PIP: Plasma membrane intrinsic proteins
TIP: Tonoplast intrinsic proteins
BZR: Brassinazole resistant
GUS: β–Glucuronidase
BCECF: 2’,7’-Bis-(2-carboxyethyl)-5-(and-6)-carboxyfluorescein
P5: Position 5
VIGS: Virus-induced gene silencing
FAZ: Fruitlet abscission zone
CEL: Cellulase
PG: Polygalacturonase
XTH: Xyloglucan endotransglucosylase/hydrolase
d: Day
Y2H: Yeast two hybrid
AZ: Abscission zone
AD: Activation domain
BD: Binding domain
QDO: A synthetic medium devoid of tryptophan, leucine, histidine, and adenine
LCI: Split luciferase complementation imaging
LUC: Luciferase
BiFC: Bimolecular fluorescence complementation
YFP: Yellow fluorescent protein
YNE: The N-terminal fragment of split YFP
YCE: The C-terminal fragment of split YFP
GST: Glutathione S-transferase
MBP: Maltose-binding protein
SDS: Sodium dodecyl sulfate
PAGE: Polyacrylamide gel electrophoresis
LC–MS/MS: Liquid chromatography tandem mass spectrometry
