# Supplementary figures and images for: LcMPK3 and LcMPK6 positively regulate fruitlet abscission in litchi
Source: Mol Hortic. 2024 Aug 6;4:29. doi: 10.1186/s43897-024-00109-z (PMC11302167; doi:10.1186/s43897-024-00109-z)

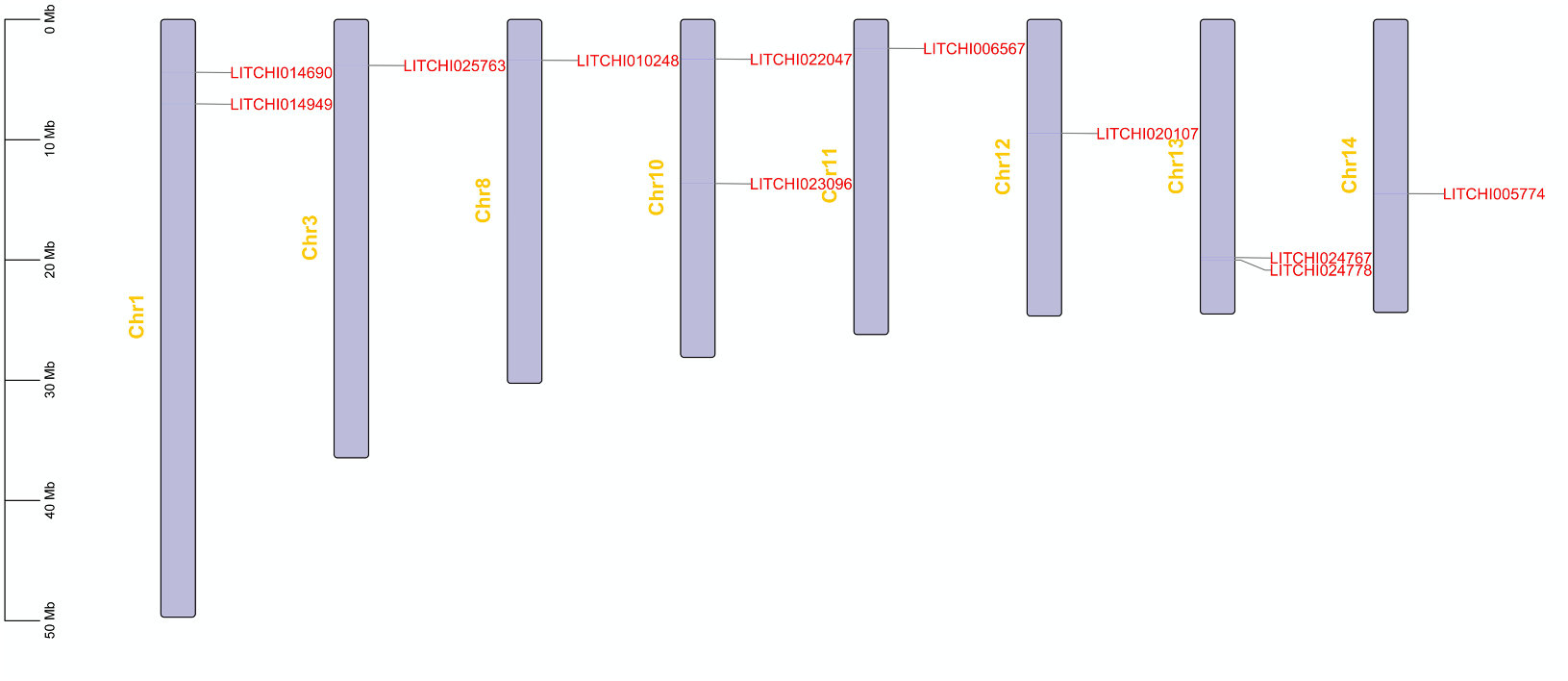

Supplement: Supplementary file 1 — Additional file 1. Figure S1. The chromosomal distribution of litchi MPKs. [file 43897_2024_109_MOESM1_ESM.png]

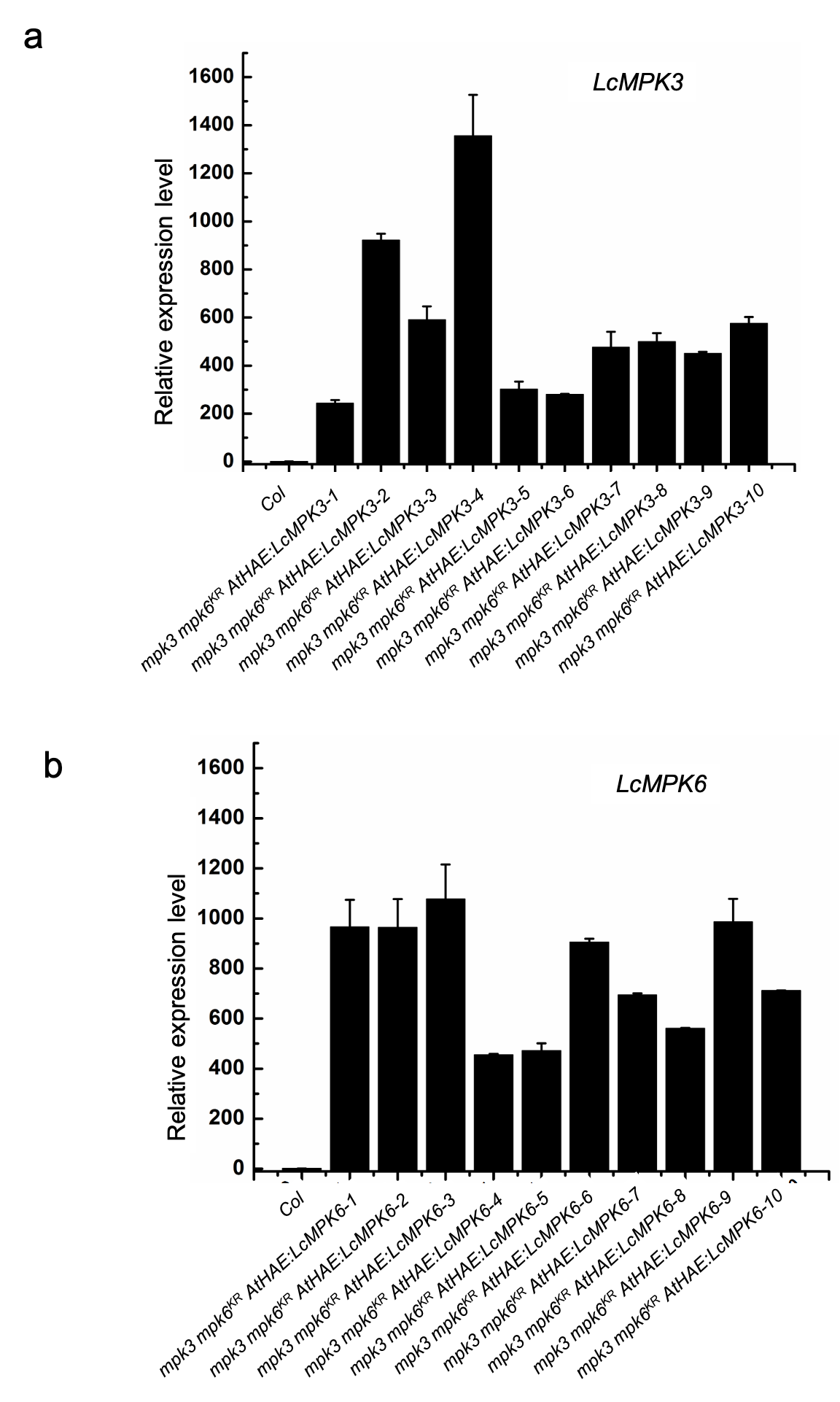

Supplement: Supplementary file 2 — Additional file 2. Figure S2. The relative expression level of LcMPK3 in the floral organ AZ of Arabidopsis mpk3 mpk6KR mutants expressing AtHAE:LcMPK3 (a), and the relative expression level of LcMPK6 in the floral organ AZ of Arabidopsis mpk3 mpk6KR mutants expressing AtHAE:LcMPK6 (b). [file 43897_2024_109_MOESM2_ESM.png]

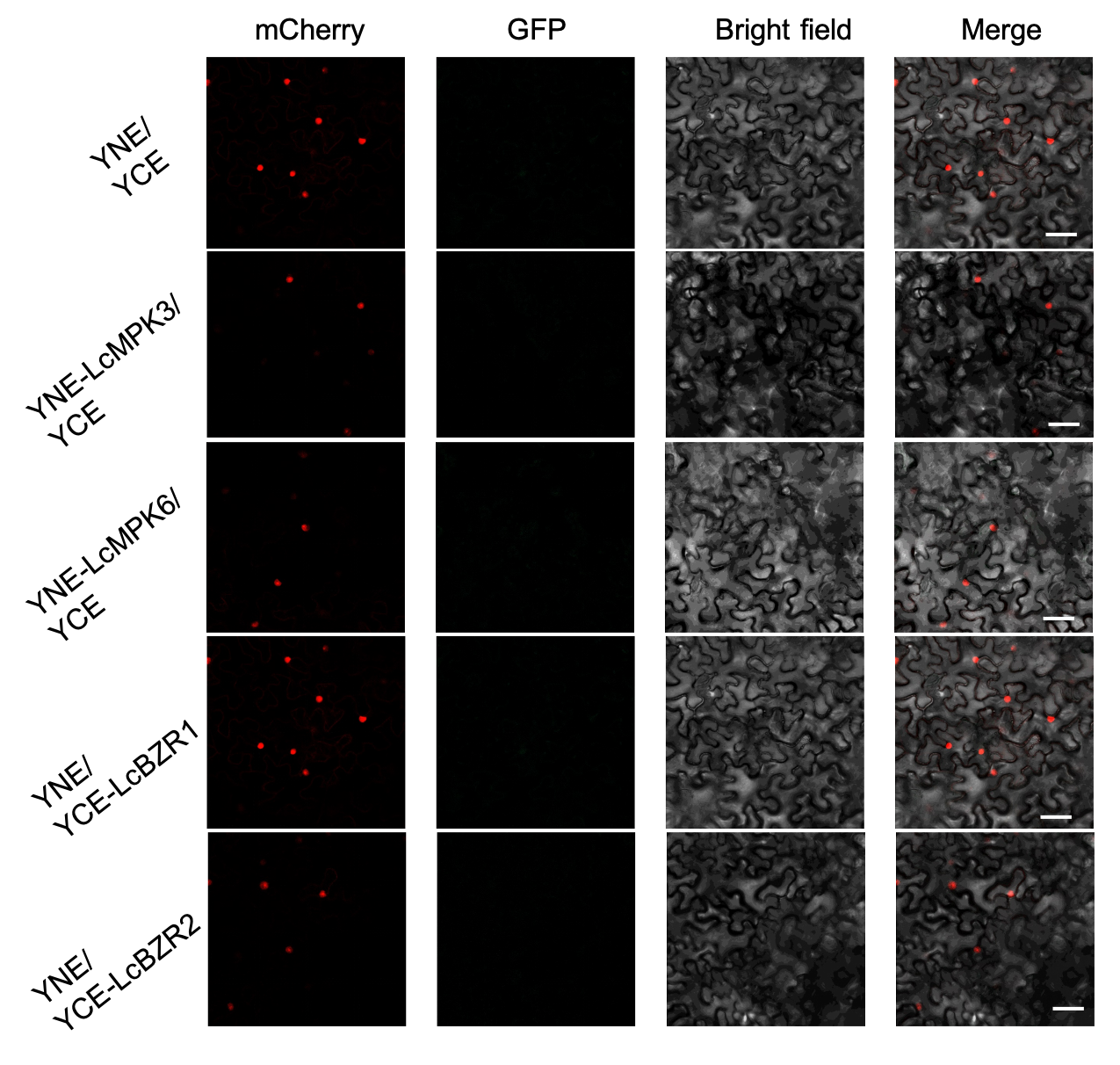

Supplement: Supplementary file 3 — Additional file 3. Figure S3. Negative controls of BiFC assays. YCE-LcBZR1 (or YCE-LcBZR2) was co-expressed with YNE, and YNE-LcMPK3 (or YNE-LcMPK6) was co-expressed with YCE. The nuclear localization signal (NLS-mCherry) and GFP fluorescence were captured using a confocal laser scanning microscope. Scale bars indicating 100 μm are shown. [file 43897_2024_109_MOESM3_ESM.png]

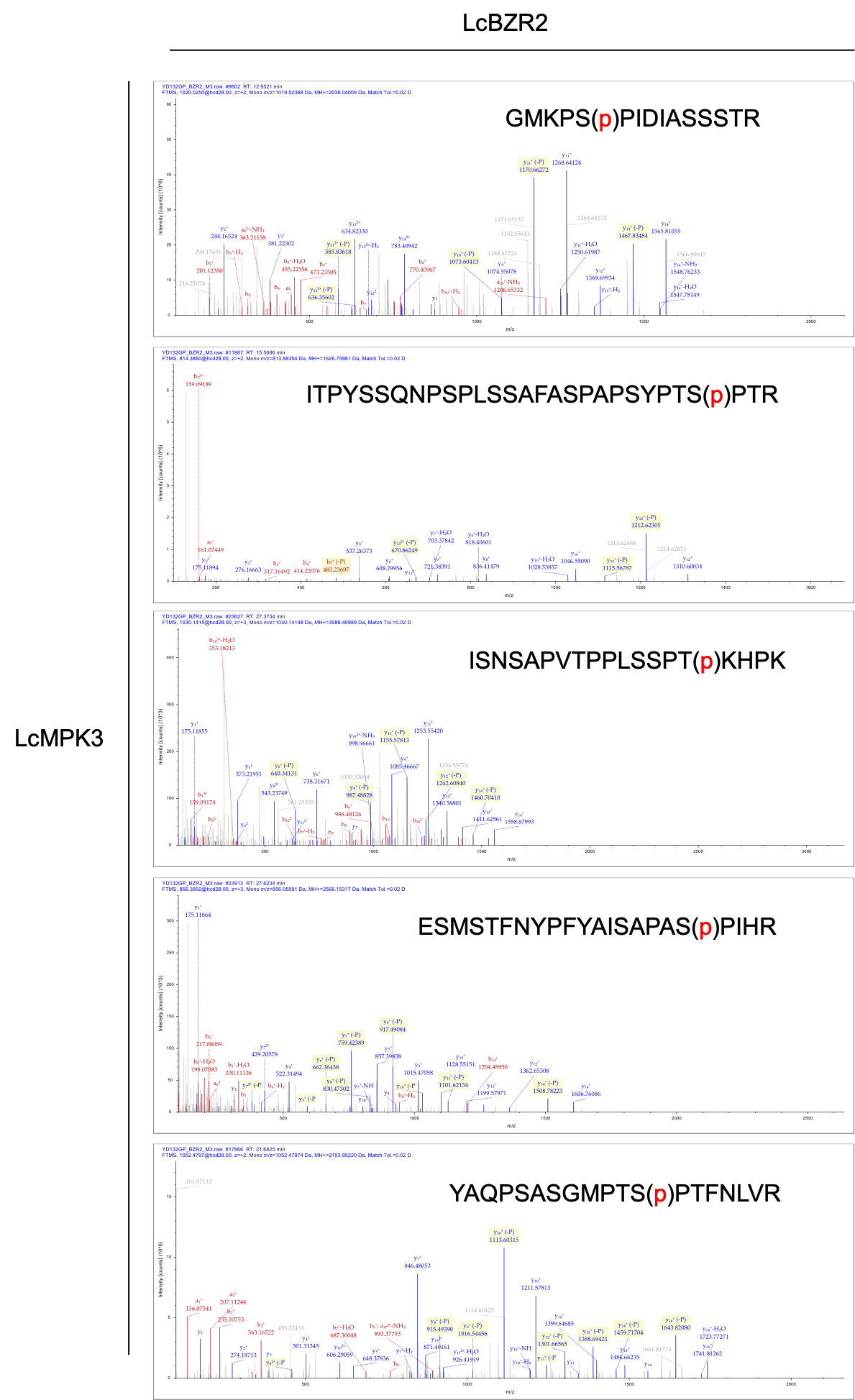

Supplement: Supplementary file 4 — Additional file 4. Figure S4. Identification of LcBZR2 sites phosphorylated by LcMAPK3 via LC-MS/MS. The mass spectrum of peptide with phosphorylation sites is shown. The b-ions and y-ions and the corresponding peptide sequence are presented, with phosphorylated serine (S) residue or threonine (T) marked by (p). [file 43897_2024_109_MOESM4_ESM.png]

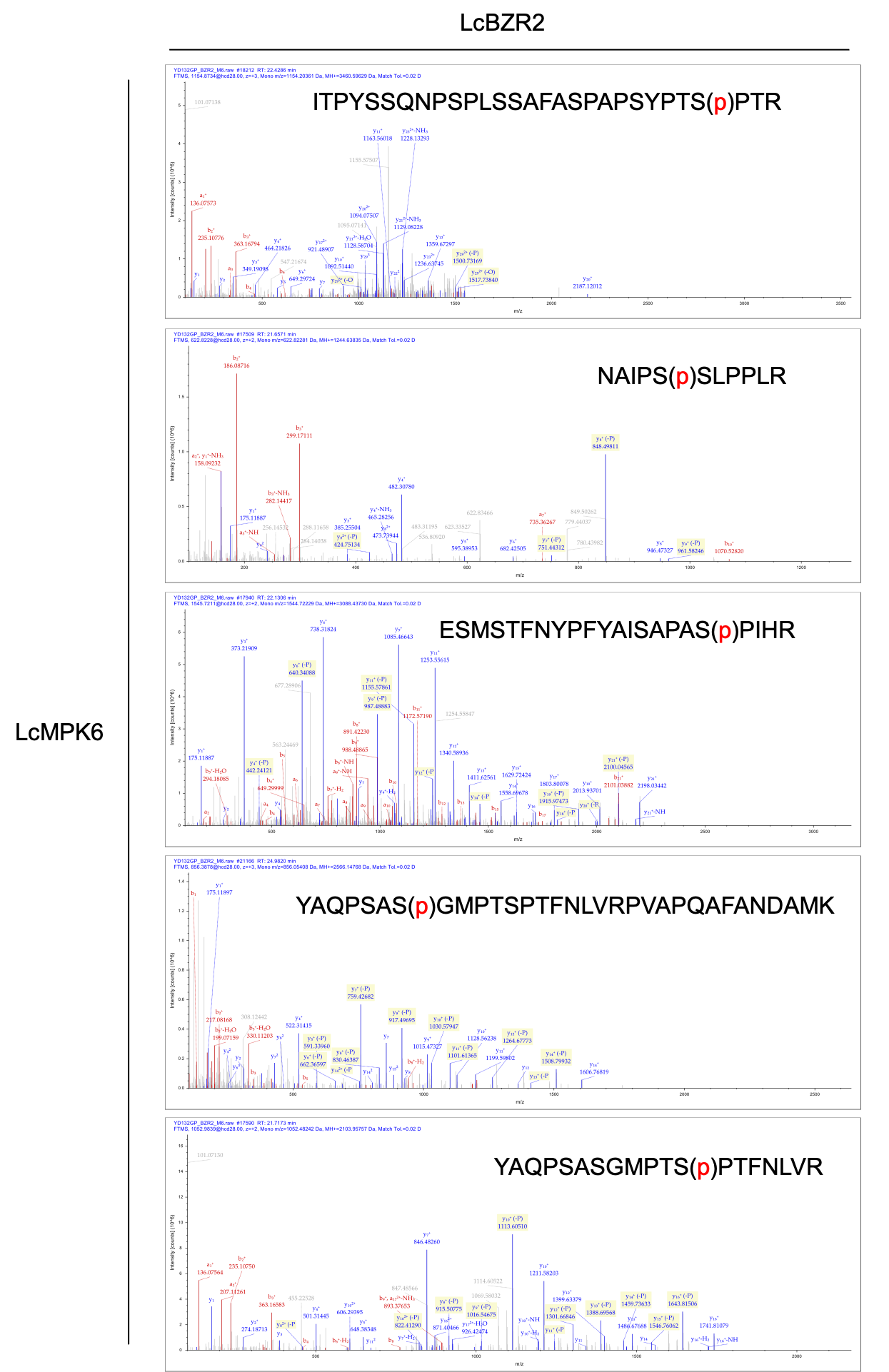

Supplement: Supplementary file 5 — Additional file 5. Figure S5. Identification of LcBZR2 sites phosphorylated by LcMAPK6 via LC-MS/MS. The mass spectrum of peptide with phosphorylation sites is shown. The b-ions and y-ions and the corresponding peptide sequence are presented, with phosphorylated serine (S) residue marked by (p). [file 43897_2024_109_MOESM5_ESM.png]
